# Lazarus1, a DUF300 Protein, Contributes to Programmed Cell Death Associated with *Arabidopsis acd11* and the Hypersensitive Response

**DOI:** 10.1371/journal.pone.0012586

**Published:** 2010-09-07

**Authors:** Frederikke G. Malinovsky, Peter Brodersen, Berthe Katrine Fiil, Lea Vig McKinney, Stephan Thorgrimsen, Martina Beck, H. Bjørn Nielsen, Stefano Pietra, Cyril Zipfel, Silke Robatzek, Morten Petersen, Daniel Hofius, John Mundy

**Affiliations:** 1 Department of Biology, University of Copenhagen, Copenhagen, Denmark; 2 The Sainsbury Laboratory, Norwich Research Park, Norwich, United Kingdom; 3 Center for Biological Sequence Analysis, Technical University of Denmark, Lyngby, Denmark; Ecole Normale Superieure, France

## Abstract

**Background:**

Programmed cell death (PCD) is a necessary part of the life of multi-cellular organisms. A type of plant PCD is the defensive hypersensitive response (HR) elicited via recognition of a pathogen by host resistance (R) proteins. The lethal, recessive *accelerated cell death 11* (*acd11*) mutant exhibits HR-like accelerated cell death, and cell death execution in *acd11* shares genetic requirements for HR execution triggered by one subclass of R proteins.

**Methodology/Principal Findings:**

To identify genes required for this PCD pathway, we conducted a genetic screen for suppressors of *acd11*, here called *lazarus* (*laz*) mutants. In addition to known suppressors of R protein-mediated HR, we isolated 13 novel complementation groups of dominant and recessive *laz* mutants. Here we describe *laz1,* which encodes a protein with a domain of unknown function (DUF300), and demonstrate that LAZ1 contributes to HR PCD conditioned by the Toll/interleukin-1 (TIR)-type R protein RPS4 and by the coiled-coil (CC)-type R protein RPM1. Using a yeast-based topology assay, we also provide evidence that LAZ1 is a six transmembrane protein with structural similarities to the human tumor suppressor TMEM34. Finally, we demonstrate by transient expression of reporter fusions in protoplasts that localization of LAZ1 is distributed between the cytosol, the plasma membrane and FM4–64 stained vesicles.

**Conclusions/Significance:**

Our findings indicate that LAZ1 functions as a regulator or effector of plant PCD associated with the HR, in addition to its role in *acd11*-related death. Furthermore, the similar topology of a plant and human DUF300 proteins suggests similar functions in PCD across the eukaryotic kingdoms, although a direct role for TMEM34 in cell death control remains to be established. Finally, the subcellular localization pattern of LAZ1 suggests that it may have transport functions for yet unknown, death-related signaling molecules at the plasma membrane and/or endosomal compartments. In summary, our results validate the utility of the large-scale suppressor screen to identify novel components with functions in plant PCD, which may also have implications for deciphering cell death mechanisms in other organisms.

## Introduction

Programmed cell death (PCD) is critical to normal growth and development as well as immune responses [1]. An intensively studied type of plant PCD is the hypersensitive response (HR), a localized cell death reaction evoked following recognition of invading pathogens [2]. This response acts to limit pathogen growth to non-infected tissues. Other defense responses include expression of pathogenesis related (*PR*) genes, production of antimicrobial phytoalexins, and systemic acquired resistance. These immune responses are activated either by receptors that recognize pathogen associated molecular patterns (PAMPs), or via direct or indirect recognition of pathogen effectors by cognate resistance (R) proteins [3]. Most R proteins contain nucleotide binding site (NB) and leucine rich repeats (LRR) domains, and either N-terminal Toll/Interleukin-1 Receptor (TIR) or coiled coil (CC) domains. In general, HR initiated via TIR R-proteins requires the regulators Enhanced Disease Susceptibility 1 (EDS1) and Phytoalexin Deficient 4 (PAD4), while HR conditioned by CC R-proteins is mediated via Non-race-specific Disease Resistance 1 (NDR1) [4]. However, disease-resistance pathways may engage more than one NB-LRR protein, and EDS1- and NDR1-dependent pathways can be activated in parallel [5], [6].

Genetic screens to identify genes required for R protein induced HR PCD have identified only few components in addition to EDS1/PAD4 and NDR1 [7]. This may be due to the overwhelming number of *R* gene alleles recovered in such screens [8], [9]. In addition to genetic analyses of R protein signaling pathways, efforts have been made to determine if PCD regulatory mechanisms are conserved between animals and plants. In animals, apoptosis and non-apoptotic forms of PCD, including necrosis and autophagy, have been described [10]. Plants lack orthologs of animal apoptotic regulators including caspases. However, caspase-like activities detected during HR cell death have been assigned to proteases including the vacuolar processing enzyme and cathepsin B [11], [12]. In addition, plants contain a complement of autophagy (ATG) effector proteins, and autophagy contributes to HR cell death conditioned by some TIR-NB-LRR R proteins [13].

Insight into HR-like PCD also comes from the characterization of mutants that exhibit *accelerated cell death* (*acd*) and defense activation in the absence of pathogen effectors. One such HR mimic, *acd11*, is a null mutant in a gene encoding a protein that facilitates sphingosine transfer between membranes *in vitro* and is homologous to mammalian glycolipid transfer proteins (GLTP) [14], [15]. Sphingolipids such as ceramide, as well as sphingoid toxins, are implicated in plant and animal PCD [16], [17]. ACD11 is also homologous to the GLTP HET-C of the fungus *Podospora anserina*. Allelic variants of *Het-c* determine incompatibility between strains, because specific combinations of *Het-c* alleles induce a PCD reaction accompanied by autophagy in asexually fusing hyphae [18]. This indicates that ACD11 homologs are involved in PCD in other organisms.

Loss of function of ACD11 is lethal, such that homozygous recessive *acd11* mutants develop normally until the 2–4 leaf stage, but then exhibit accelerated cell death similar to that seen in lesion-mimic mutants [2]. PCD in *acd11* is dependent upon the phytohormone salicylic acid (SA), and can be suppressed by expression of a bacterial SA hydroxylase (*nahG*), or by mutations in *PAD4* and *EDS1* [15], [19]. *acd11/nahG* is reversed to the *acd* phenotype by application of the SA agonist benzo(1,2,3)thiadiazole-7-carbothioic acid-*S*-methyl ester (BTH).

Here, we use this system to develop an exhaustive genetic screen for suppressors of *acd11*-related cell death which we call *lazarus* (*laz*) mutants. We show that this screen is useful to identify regulators of plant cell death, and to further describe the pathway(s) engaging the resistance regulators EDS1/PAD4. Map-based cloning of *laz1* identified a conserved, six transmembrane DUF300-containing protein, homologous to the human tumor suppressor TMEM34 and to the Ost-α component of the transporter of organic solutes Ost-α/Ost-β. LAZ1 contributes to EDS1-dependent HR cell death conditioned by the TIR R protein RPS4, and to HR PCD initiated by the CC R protein RPM1 in a partly NDR1-dependent manner. This implicates LAZ1 in *acd11* related cell death and as a more general component required for PCD associated with the HR.

## Results

### Isolation of *laz* mutants

**Figure 1:**
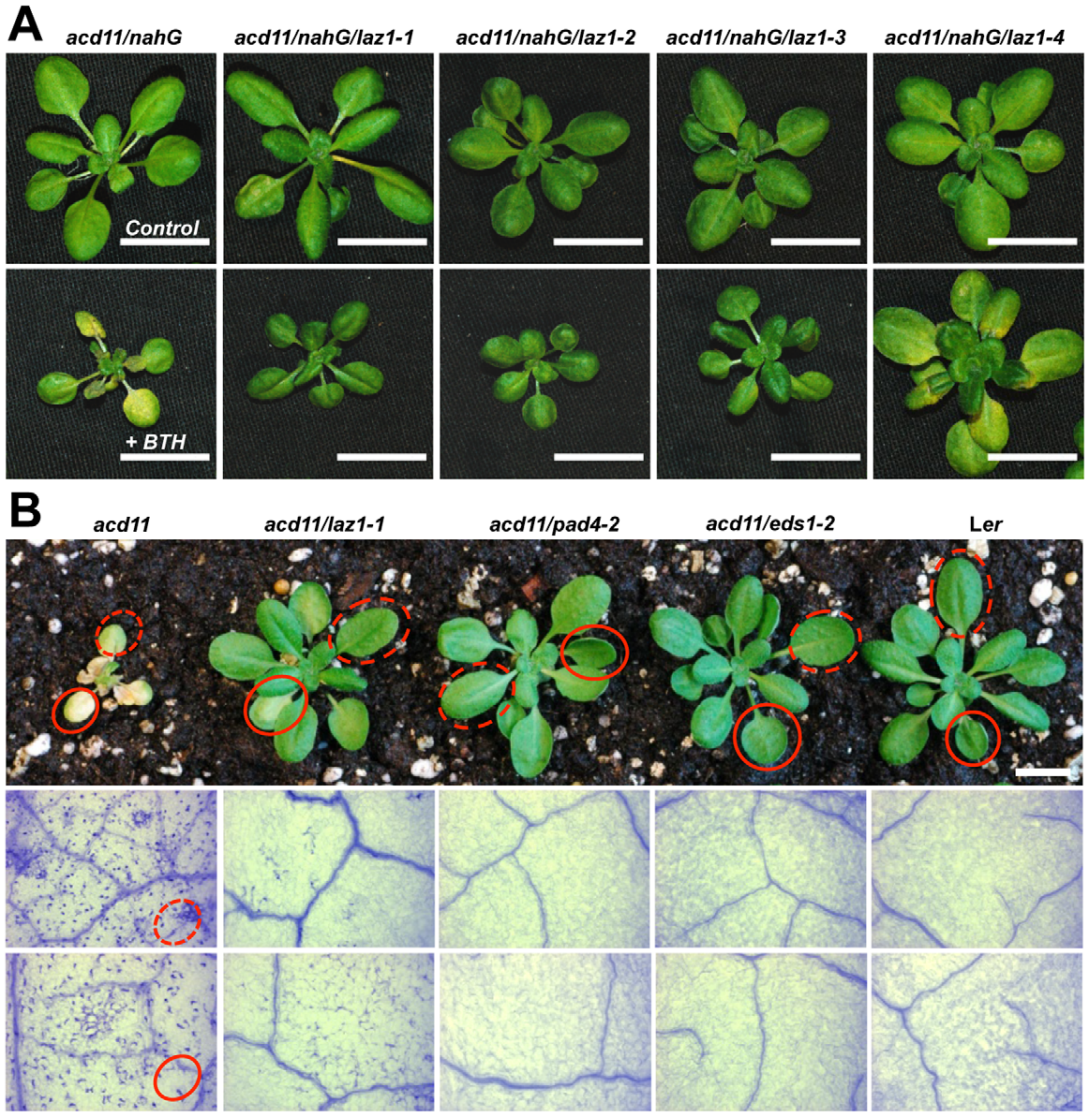
*laz1*-mediated suppression of cell death in *acd11*. (A) Suppression of BTH-induced *acd11* cell death in the four *laz1* alleles in comparison to the *acd11*/*nahG* background. Two-week-old plants were sprayed with 100 µM BTH and photographed 7 days after treatment. Untreated control plants are shown in the upper panel and BTH treated plants in the lower panel. Scale bars represent 1 cm. (B) Suppression of cell death in *acd11* after out-crossing the *nahG* transgene. *acd11/laz1-1* plants were grown under SD for four weeks and compared to *acd11*, *acd11/eds1-2*, *acd11/pad4-2* and wild type L*er* (upper panels). Trypan blue staining (lower panels) of younger (dashed circles) and older (solid circles) leaves revealed numerous dead cell foci in *acd11* compared to their marked reduction in *acd11/laz1*, and apparent absence in *acd11/eds*, *acd11/pad4* and wild type L*er*. Bar indicates 1 cm.

BTH treatment of *acd11/nahG* leads to growth arrest and cell death induction in all tissues (Figure 1A) [14], [15]. We therefore screened for suppressor mutants in M2 populations of ethyl-methane-sulphonate (EMS), di-epoxy-butane (DEB) and γ-irradiation mutagenized *acd11/nahG* following BTH treatment. More than 200 putative mutants were identified among ∼3 million M2s screened. Analyses of inheritance and allelism among 50 mutants identified five alleles of *eds1* and 7 of *pad4*, validating the utility of the screen (Table S1). The remaining 38 fell into complementation groups of 11 recessive and 2 dominant suppressor loci here called *laz* mutants in reference to the biblical resurrection of Lazarus. *laz* mutants were not specifically defective in BTH uptake or response, because they also suppressed cell death induced by the chemically distinct SA agonist, isonicotinic acid (data not shown).

One of the *laz* mutants that showed significant suppression of the cell death response to BTH in the *acd11 nahG* background was the γ-irradiation induced, recessive *laz1-1*. Test crosses showed that *laz1-1* was allelic to three other recessive mutants. *laz1-1*, *laz1-2* and *laz1-3* exhibited similar suppression of *acd11* cell death (Figure 1A). *laz1-4* suppression appeared weaker, which may be due to the nature of this mutation as discussed below. Notably, in addition to the weaker cell death suppression, *laz1-4* plants were larger than the other three alleles. Whether this difference in growth is due to the *laz1-4* mutation or to a second site mutation is unknown.

Upon out-crossing the *nahG* transgene, *laz1-1* still suppressed cell death in *acd11*, confirming that *LAZ1* is required for cell death, and not for BTH responsiveness (Figure 1B). However, *laz1-1* does not fully suppress cell death as indicated by the presence of trypan blue stained dead cells in older leaves compared to complete suppression by *eds1* and *pad4* in the absence of BTH treatment. Nonetheless, loss of function *laz1* mutations suppress *acd11*-dependent cell death such that *acd11/laz1* double mutants survive throughout development to flower and set seed, in marked contrast to the fate of *acd11* (Figure S1).

### Transcript profiling of *laz1-1* reveals suppression of the *acd11* phenotype

**Figure 2:**
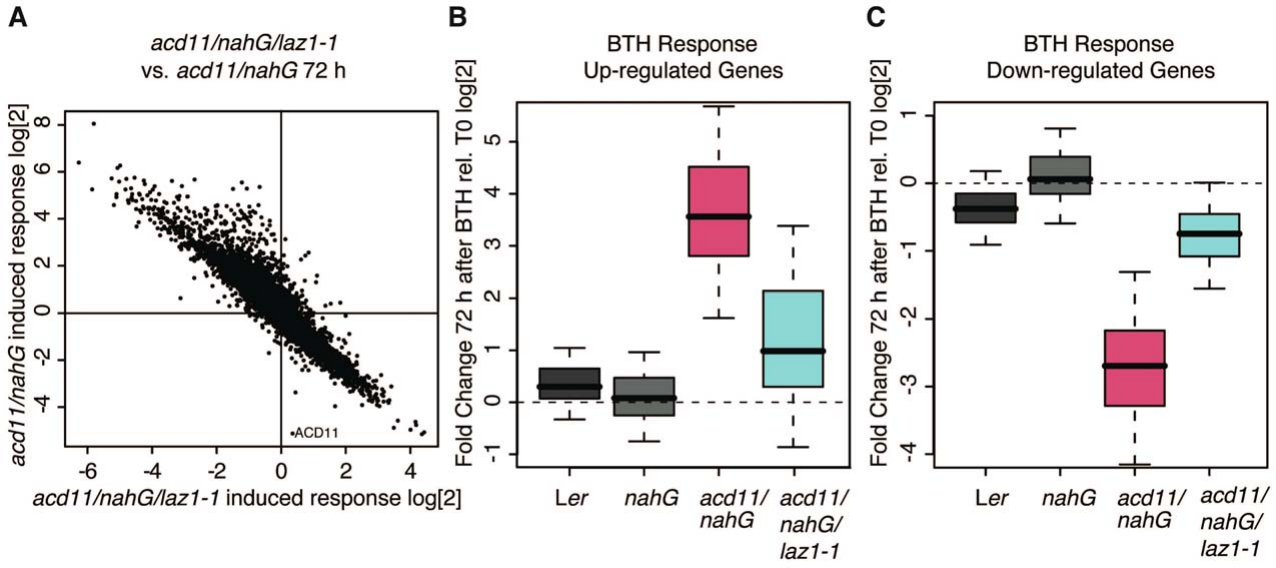
Transcriptome analysis of *laz1*. (A) Comparison of the global expression fold change between *acd11/nahG* and *nahG* with the fold change between *acd11/nahG/laz1-1* and *acd11/nahG* 72 hr after BTH treatment. This showed a strong opposite response with a Pearson correlation of −0.93. (B, C) Box plots of the expression of the 500 most significantly up- (B) or down-regulated (C) genes in BTH-treated *acd11/nahG*. The genes exhibit only moderate responses in *acd11/nahG/laz1* plants.

Affymetrix ATH1 GeneChip arrays were used to obtain global transcript profiles from L*er* wild type, *nahG* and *acd11*/*nahG* plants at 0, 12, 24, 72 and 120 hr after BTH treatment. This showed that significantly responding genes in *acd11/nahG* were preferentially up- or down-regulated by 72 hr after BTH treatment compared to the *nahG* control (data not shown). To assess the overall effects on gene expression caused by the *laz1-1* allele in the *acd11* background, total mRNA was isolated from *acd11/nahG/laz1-1* plants before and 72 hr after BTH treatment. We then compared the global expression fold change between *acd11/nahG* and *nahG* with the fold change between *acd11/nahG/laz1-1* and *acd11/nahG* at 72 hr. This detected strong differential gene expression after BTH treatment in *acd11/nahG* compared to *nahG,* which represents the ‘*acd11* response’. The ‘*laz1-1* response’ was obtained by comparing *acd11/nahG/laz1-1* to *acd11/nahG*. Interestingly, genes strongly induced or repressed in the ‘*acd11* response’ were either nonresponsive or only weakly responsive in *acd11/nahG/laz1-1*. This can be shown as a strong inverse ‘*acd11* response’ in the *acd11/nahG/laz1-1* to *acd11/nahG* comparison, which is illustrated by the negative Pearson correlation of −0.93 in a fold change scatterplot (Figure 2A).

In keeping with this correlation, the 500 most significantly differentially expressed genes in BTH treated *acd11/nahG* plants at 72 hr showed very moderate responses in *acd11/nahG/laz1-1* plants (Figure 2B–C). This indicates that *laz1*-mediated PCD suppression is already manifested by reversion of the transcriptomic phenotype in *acd11/nahG* prior to the occurrence of cell death symptoms (Figure 1). Transcript levels of the *acd11* suppressors EDS1 and PAD4 were comparable in *acd11/nahG* and *acd11/nahG/laz1-1* upon BTH treatment (Figure S2). This indicates that EDS1 and PAD4 do not function downstream of LAZ1. In addition, other marker genes involved in defense and death were investigated, and some showed either complete suppression or an intermediate response in *acd11*/*nahG*/*laz1-1* compared to *acd11/nahG* (Figure S2).

### Map-Based cloning of *LAZ1*

**Figure 3:**
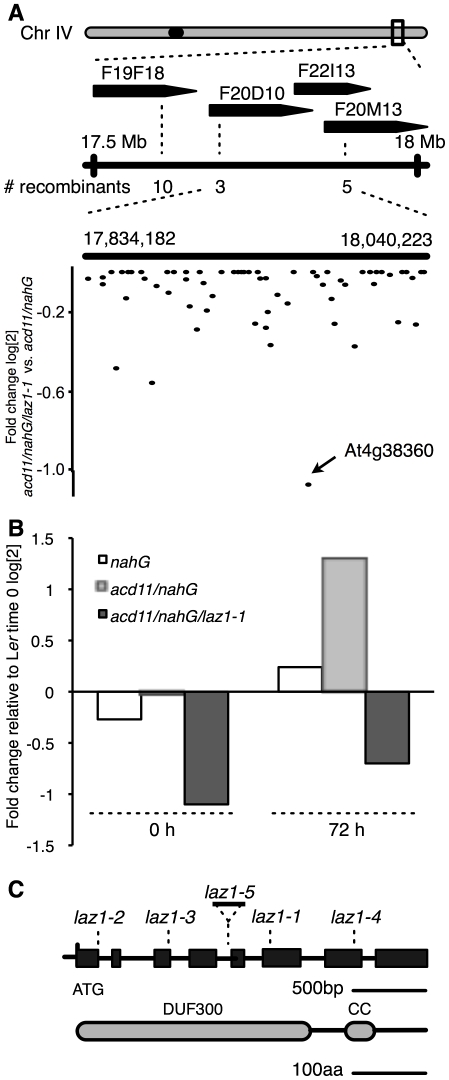
Map-Based Cloning of *LAZ1*. (A) Map of the *LAZ1* locus on chromosome 4. Positions of markers on three BACs for mapping *laz1-1* to a 200 kb interval are indicated (see Table S2 for primers) with the numbers of recombinants. Lower panel shows differential expression of genes within the interval measured by transcript profiling. This identified At4g38360 as the gene in the interval with the most reduced expression between *acd11/nahG/laz1-1* and *acd11/nahG* (log2 fold change with expression intensity ceiling set to 0). The probe set for At4g38360 is indicated. (B) Expression profiling of At4g38360 depicted relative to L*er* before treatment (0 hrs). As determined by 2-way ANOVA with the factors treatment and genotype, At4g38360 transcripts accumulated (p-value = 0.018) in *acd11/nahG* upon BTH treatment (72 hrs), but were not induced in *acd11/nahG/laz1-1* or background controls. Similarly, At4g38360 was significantly induced in the *acd11* mutant (*p* = 0.00059; determined by t-test, data not shown). (C) Structure of *LAZ1* including positions of the *laz1* mutations identified in the screen and the T-DNA integration site of *laz1-5* (SALK_034193). The coding region and protein domains (DUF300, domain of unknown function 300; CC, coiled-coil) are boxed. *laz1-1* is a γ-induced, one-base pair deletion in exon 6 leading to a premature stop codon (see Figure S3A). *laz1-4* has an EMS-derived G->A resulting in conversion of a plant conserved Aspartate to Asparagine (D360N). The base pair changes in DEB induced *laz1-2* (T->A) and EMS-induced *laz1-3* (G->A) lead to mutations in splice donor sites of introns 1 and 3, causing deletions in preceeding exons (see Figure S3B–C).

To identify the *LAZ1* locus, a mapping population was generated by crossing *acd11/nahG/laz1-1* (ecotype L*er*) to *acd11-2/nahG* in the Col-0 ecotype [15]. The *γ-*mutagenized *laz1-1* allele was used because irradiation induced deletions might lead to changes in *laz1-1* mRNA levels detectable by the transcript profiling described above. Genetic markers polymorphic between L*er* and Col-0 were used to map *laz1-1* to a 200 kb interval on the lower arm of chromosome 4 (Figure 3A). The gene in this interval with the most reduced expression in *laz1-1* was sequenced. This identified a 1bp deletion in exon 6 in the most repressed gene (At4g38360; Figure 3A) resulting in a frameshift and a premature stop (Figure S3A). Interestingly, the microarray data showed that *LAZ1* transcripts accumulate upon BTH treatment in *acd11*/*nahG* plants, but not in *nahG* or *acd11/nahG/laz1-1* (Figure 3B). Furthermore, in *acd11*, *LAZ1* transcripts accumulate to levels that are almost three times higher than in wild type (microarray data not shown).

Sequencing of the three other *laz1* alleles revealed that all had mutations in At4g38360. These included a DEB induced T->A transversion (*laz1-2*) and two EMS induced G->A transitions (*laz1-3* and *laz1-4*), leading to disrupted intron splice donor sites and subsequent deletions in transcripts of *laz1-2* and *laz1-3* (Figure S3B–C), as well as the conversion of aspartate 360 to asparagine (D360N) in the weaker *laz1-4 allele.* Although the D360N mutation is not within a predicted transmembrane segment (Figure 3C and S4), the suppression by *laz1-4* may be due to reduced function and/or mislocation of the mutated LAZ1 protein (see below). Based on these findings, we conclude that *LAZ1* corresponds to the At4g38360 locus.

### *laz1* suppresses RPS4 and RPM1 conditioned HR cell death

**Figure 4:**
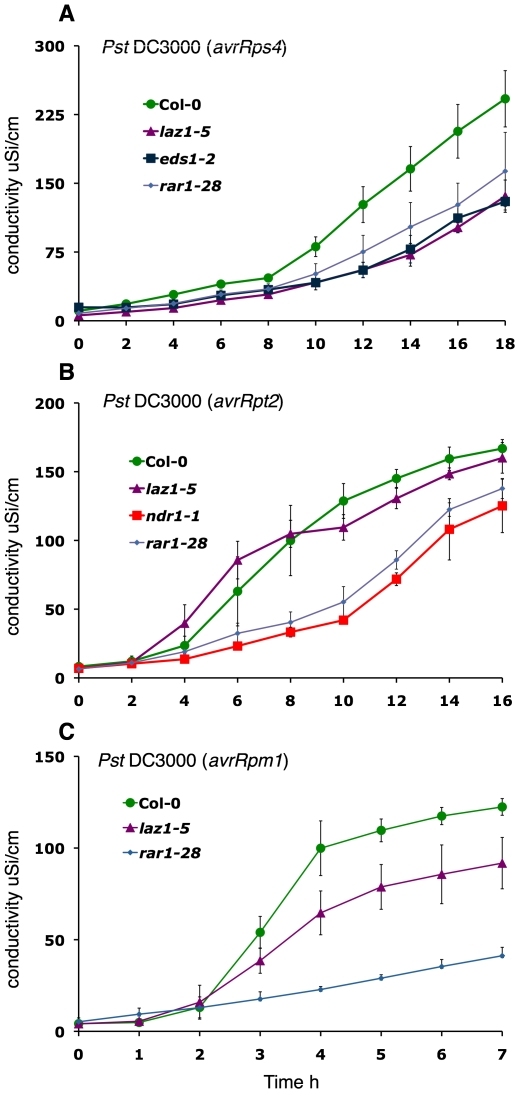
*laz1 s*uppression of HR cell death. Ion leakage assays, with mean and standard errors (SE) in A–C calculated from four disks per treatment with four replicates within an experiment, after: (A) *Pto* DC3000 (*avrRps4*) infection of *laz1-5* compared to Col-0, *eds1-2* and *rar1-28*. (B) *Pto* DC3000 (*avrRpt2*) infection of *laz1-5* compared to Col-0, *ndr1-1* and *rar1-28*. (C) *Pto* DC3000 (*avrRpm1*) infection of *laz1-5* compared to Col-0 and *rar1-28*.

To investigate the function of LAZ1 in induction of HR cell death, we challenged a *laz1* T-DNA insertion mutant, here termed *laz1-5*, in ecotype Col-0 [20] (Figure 3C, S5A-C) with the bacterial pathogen *Pseudomonas syringae* pv. tomato (*Pst*) DC3000 harboring the effector AvrRps4. This effector is recognized via the TIR-NB-LRR resistance (R) protein RPS4, which triggers the HR via an EDS1/PAD4 dependent pathway which is also required for cell death in the *acd11* mutant. The levels of HR cell death upon infection can be quantified by electrolyte leakage measurements, as cell death causes electrolyte release measured as changes in the conductance of a bath solution [21]. The *laz1-5* mutation suppressed the extent of cell death elicited by *Pst* DC3000 (*avrRps4*) to comparable levels as the enhanced susceptibility *eds1* and *rar1* controls which are either fully impaired or compromised in RPS4-conditioned HR [4], [22] (Figure 4A). This indicates that LAZ1 may be involved both in the induction of EDS1/PAD4 mediated HR and in accelerated cell death in the *acd11* mutant.

To test whether LAZ1 is also involved in HR reactions with other genetic requirements, we checked the effects of *laz1-5* on the EDS1/PAD4 independent HR cell death induced by *Pst* DC3000 (*avrRpt2*). The defense reaction induced against this effector occurs exclusively via NDR1. In this case, *laz1-*5 exhibited ion leakage levels comparable to wild type (Figure 4B). This indicates that LAZ1 is not required for HR induction elicited through pathways exclusively dependent on CC-NB-LRR resistance proteins.

We also investigated the role of LAZ1 in HR induced by the partially NDR1 dependent effector AvrRpm1. Like AvrB, AvrRpm1 is recognized by the CC-NB-LRR R protein RPM1, although one or more TIR-type R proteins also contribute to HR induction via RPM1 [5]. As expected, wild type Col-0 plants exhibited high levels of electrolyte leakage, while the *laz1-5* mutation suppressed the extent of cell death, but not to the level of the *rar1* mutant, which is fully impaired in RPM1 conditioned HR [22] (Figure 4C). Collectively, these data demonstrate that LAZ1 contributes to the induction or execution of cell death in *acd11*, as well as to both EDS1/PAD4 dependent and partially NDR1 dependent HR induced in response to pathogen effectors. Furthermore, it confirms that independent pathways lead to HR cell death [13] and at least one of these is dependent on LAZ1.

Given that LAZ1 is mainly involved in the EDS1-dependent pathway of pathogen-triggered HR, we investigated whether *laz1* abolished TIR-NB-LRR R protein-mediated resistance in a similar manner as *eds1*. Bacterial growth assays with Pst DC3000 (*avrRps4*) revealed that RPS4-mediated resistance was not compromised in *laz1-5* mutant plants (Figure S6A). Similarly, *laz1*-5 mutants showed wildtype-like responses towards syringe or spray inoculation with virulent *Pst* DC3000 (Figure S6B). These results indicate that LAZ1 function in HR cell death execution is dispensable for induced resistance and that LAZ1 does not seem to play a significant role in basal defence responses.

### LAZ1 topology and orientation

**Figure 5:**
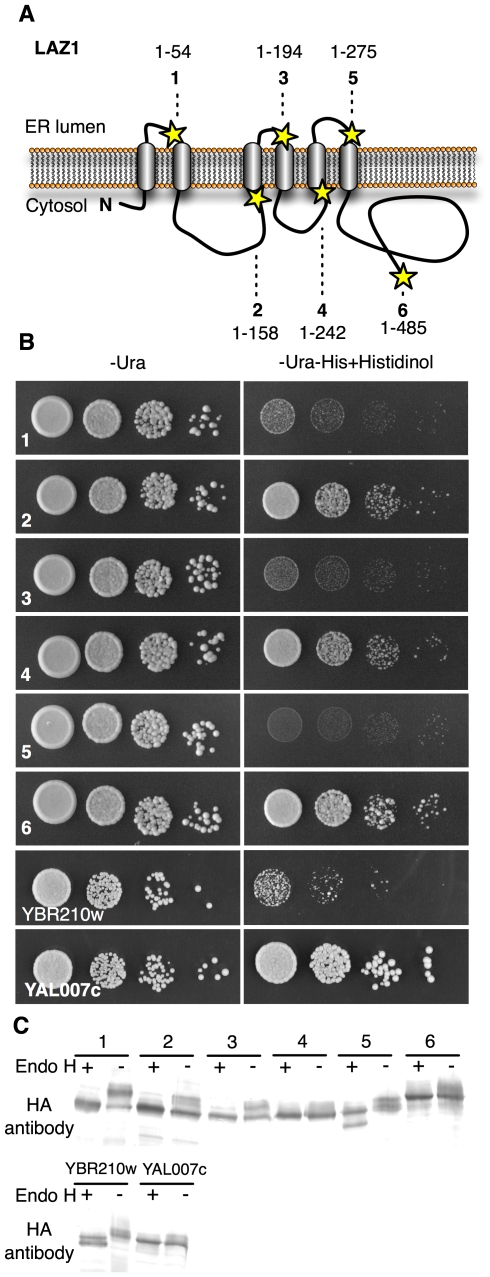
LAZ1 topology. (A) Six LAZ1 forms (1–6) were C-terminally fused to the dual SUC2/HIS4C reporter to determine their orientation in membranes. These LAZ1 fusions were chosen to assess the validity of predicted transmembrane regions (Figure S4). Lengths and positions of the fusions are indicated by lines and stars, respectively. (B) Growth of yeast harboring the six LAZ1 reporter fusions on histidinol containing medium indicates cytosolic localization of the reporter due to its histidinol dehydrogenase activity. YBR210w (ER localized C-terminus) and YAL007c (cytosolic localized C-terminus) served as controls [24]. (C) The glycosylation status of six LAZ1 fusions (Fig. 5A) was determined by comparing the sizes of endoglycosidase H (endo H) treated and untreated samples followed by Western blotting using anti-HA antibody. Faster migrating (lower) bands after endo H treatment indicate that the fusion is glycosylated and the SUC2/HIS3 C-terminal reporter resides in the ER. YBR210w (ER localized C-terminus) and YAL007c (cytosolic localized C-terminus) served as controls [24].

LAZ1 encodes a protein of unknown function with a conserved, N-terminal DUF300 domain. It is predicted to be localized to the endomembrane system, and to have 5–7 transmembrane regions [23], 4 of which can be aligned to predicted membrane spanning segments in the closest LAZ1 human homolog TMEM34 (Figure S4, S7A). Based on these predictions, we assessed the topology of LAZ1 and TMEM34 using yeast-based assays with full-length and truncated versions of the proteins C-terminally fused to the dual SUC2/HIS4C reporter [24] (Figure 5, S7). This monitors the location of the C-terminal reporter fusion relative to the ER membrane. The SUC2 reporter region contains N-glycosylation sites which can be heavily glycosylated in the ER lumen. The His4C region encodes a cytosolic histidinol dehydrogenase permitting growth on media without histidine but with histidinol. The assays showed that strains harboring fusions to full-length LAZ1 and LAZ1 truncations comprising amino acids 1–158 (LAZ1_1–158_) or 1–242 (LAZ1_1–242_) grew on selective media (Figure 5A–B), indicating the cytosolic localization of their C-termini. In contrast, strains harboring LAZ1_1–54_ LAZ1_1–194_ and LAZ1_1–275_ reporter fusions did not grow, indicating their C-termini were in the ER lumen. Similar assays were performed with five fusions of full-length and truncations of TMEM34 (Figure S7B–C). Notably, the strain harboring TMEM34_1–49_ did not grow on selective media, while those harboring TMEM34_1–80_, TMEM34_1–99_, and TMEM34_1–171_ grew well. These results, in conjunction with the theoretical topology models (Figure S4, S7A), suggest that both LAZ1 and TMEM34 contain six membrane spanning regions within their DUF300 domains, have N- and C-termini in the cytosol, and contain cytosolic regions of ∼100 residues between their 2^nd^ and 3^rd^ membrane spanning regions.

These models were verified by assaying the glycosylation of the fusions by comparing the mobility of endoglycosidase H treated and untreated samples. This showed that while the LAZ1_1–54_, LAZ1_1–194_ and LAZ1_1–275_ fusions were glycosylated, the LAZ1_1–242_ and full-length LAZ1 fusions were not (Figure 5C). Although the LAZ1_1–158_ fusion appeared to be partially glycosylated, the robust growth of yeast harboring it, and the confirmatory evidence for the orientation of the adjacent transmembrane regions for both LAZ1 and TMEM34 (Figure 5B, S7B–C) suggest that LAZ1 and TMEM34 share a common topology with cytosolic N- and C-termini. These preliminary results provide a basis for further structure/function analyses of DUF300 proteins.

### LAZ1 localisation

**Figure 6:**
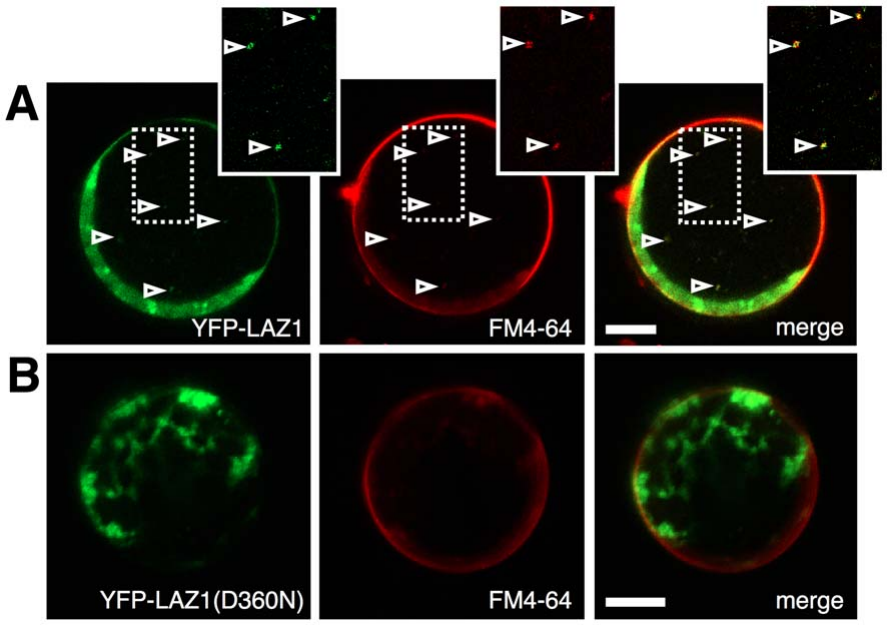
Subcellular localisation of LAZ1. (A) Confocal images of *Arabidopsis* mesophyll protoplast detect the distribution of YFP-LAZ1 between cytosol, plasma-membrane and vesicle-like structures. YFP-LAZ1 localisation at plasma membrane and vesicle compartments (arrows) was confirmed by co-staining with the membrane selective dye FM4–64. Enlargement of boxed region highlights the overlap between YFP-LAZ1 and FM4–64 staining in endosomal compartments. Size bar corresponds to 10 µm. (B) YFP-LAZ(D360N) localization is primarily cytosolic and shows no overlap with FM4–64 staining. Size bar represents 10 µm.

To investigate the localization of LAZ1 protein *in planta*, a reporter construct with yellow fluorescence protein (YFP) fused to the N-terminus of LAZ1 under the control the constitutive CaMV 35S promoter was transiently expressed in mesophyll protoplasts. Confocal microscopy revealed that YFP-LAZ1 is distributed between the cytosol, plasma membrane and mobile vesicle-like structures that co-stained with FM4–64 (Figure 6A) [25], [26]. Comparable localisation patterns were also detected upon transfection with a construct carrying a C-terminally fused red fluorescence protein (RFP) reporter (LAZ1-RFP) (data not shown). This indicates that the N-terminal YFP tag does not interfere with potential targeting motifs. In addition, the detection of fluorescence from both N- and C-terminal tags confirms that the N- and C-termini are exposed to the cytosol (Figure 5).

We then analyzed whether cell death suppression in *laz1-4* may be due to altered subcellular localisation of the LAZ1 protein by expressing a YFP-LAZ1(D360N) fusion protein in protoplasts. Notably, microscopic analysis showed that this mutant fusion was primarily cytosolic with little signal in the plasma membrane and no co-localisation with FM-64 vesicles (Figure 6B). This altered localisation of the point mutant variant may explain, at least in part, its suppression of *acd11* mediated cell death. However, since the mutated amino acid residue (D360N) is predicted to be localized in a cytoplasmic domain and not in any targeting motif (Figure S4), the potential link between mislocation and putative transporter functions of LAZ1 at the plasma membrane and/or endosomal compartments remains to be determined.

## Discussion

Our screen for regulators of plant PCD identified *LAZ1* (At4g38360). Loss of LAZ1 function suppressed cell death in the *acd11* mutant, as well as certain types of HR cell death. LAZ1 belongs to a family of DUF300 transmembrane proteins conserved among eukaryotes. We provide evidence for similar topologies for LAZ1 and the human DUF300 protein TMEM34, and show that LAZ1 functions in the regulation or execution of PCD.

### LAZ1 in PCD associated with R protein dependent HR

*LAZ1* contributes to cell death activated in *acd11* or mediated by the TIR-NB-LRR protein RPS4 upon avirulent pathogen infection. Our transcriptome analysis revealed that BTH induced levels of *EDS1* (and *PAD4*) mRNAs are not affected by *laz1* loss-of-function (Figure S2), suggesting that LAZ1 contributes to the execution or regulation of *acd11* cell death downstream of EDS1. The potential redundancy among LAZ1 homologs (At1g77220, 55% amino acid identity; At1g23070, 42% identity; Figure S4) may explain the weaker suppression of *acd11* by *laz1* compared to *eds1*, as EDS1 may engage other pathways to regulate or execute cell death. We also found that LAZ1 contributes to HR PCD initiated by the partially NDR1-dependent CC-NB-LRR protein RPM1, whereas HR conditioned by the strictly NDR1-dependent CC-NB-LRR protein RPS2 did not engage *LAZ*1 function.

CC- and TIR-type NB-LRR R proteins function additively in disease resistance responses [5], [6]. In the case of AvrB-triggered RPM1-dependent resistance, the TIR-NB-LRR protein TAO1 is required for mounting a full response, while TAO1 is not required for induction of AvrRpm1-triggered, RPM1-dependent responses [5]. These findings suggest that an unknown TIR-NB-LRR contributes to AvrRpm1-triggered resistance, a model supported by the HR suppression observed in *laz1* upon infection with *Pst* DC3000 *avrRps4* and *avrRpm1*, but not *avrRpt2*. This implicates LAZ1 in cell death reactions partly or fully conditioned by TIR-dependent signaling. In addition, HR cell death triggered by TIR-NB-LRRs via EDS1 requires autophagy, whereas HR initiated by CC-type R proteins via NDR1 is either autophagy-independent or engages autophagic components with cathepsins and other unidentified cell death mediators [13]. These data separate EDS1 and NDR1 conditioned HR cell death both at the initiation and execution stages. However, we note that the contribution of LAZ1 to TIR-dependent cell death does not impact induced resistance to avirulent bacteria carrying AvrRps4 (Figure S6A), supporting a potential separation of HR defence and death responses as seen in *dnd* (defence, no death) mutants [27], [28]. Thus, together with the finding of wild-type like responses of *laz1-5* mutants to virulent bacterial infection (Figure S6B), a primary involvement of LAZ1 in defence-independent cell death execution processes may be proposed.

### Pro-death function(s) of LAZ1 and a human homolog

HR cell death is strictly regulated to avoid inappropriate induction during normal growth, and to avoid unrestricted cell death in response to infection. The clear effect of LAZ1 expression on the induction or execution of cell death indicates that LAZ1 expression should be tightly regulated. This is consistent with our demonstration that *LAZ1* mRNA accumulates in *acd11/nahG* upon BTH treatment, as well as developmentally in senescent tissue [29].

The closest human LAZ1 homolog, TMEM34, is implicated as a tumor suppressor because TMEM34 expression is reduced in anaplastic thyroid cancer cell lines, and transfection of TMEM34 into cancer cells leads to cell growth inhibition [30]. In addition, our topology assays suggest that TMEM34 and LAZ1 have similar overall structures that may imply analogous functions. Thus, it is tempting to speculate that LAZ1 and TMEM34 represent phylogenetically distant DUF300 proteins with functions in PCD across the eukaryotic kingdoms. Nevertheless, a direct role of TMEM34 in PCD regulation and/or execution, as demonstrated here for LAZ1, remains elusive.

### LAZ1 may be a transporter of bile acid-like compounds

The biochemical function of LAZ1 may be related to that of the human DUF300 homolog Ost-α, which is part of the Ost-α-Ost-β transporter responsible for secretion of bile acids and related compounds into the portal blood circulation [31]. Interestingly, the primary animal bile acid cholic acid (CA) triggers HR cell death and defense responses in rice [32]. Comparison of elicitor activity with other bile acid compounds revealed specific recognition of CA, suggesting that CA induces the HR by mimicking an endogenous plant signal. Bile acids have not been found in plants, but are structurally similar to brassinosteroids. These phytohormones are involved in developmental and growth processes, and in modulating defense responses [33]. Notably, certain brassinosteroids (25-hydroxy-24-epibrassinolide, 25-hydroxy-3,24-epibrassinolide) may fulfill some of the structural requirements to be perceived by the same receptors or transporters as CA. Thus, future study may clarify whether such brassinosteroids have death-promoting functions in plants and can be transported or modulated by LAZ1.

In this context, we note that LAZ1 contains putative endocytic sorting motifs [34] (Figure S4) similar to those in the mouse endosomal DUF300 protein Sdmg1 [35]. In addition, our subcellular localization studies detected LAZ1 localization not only in the cytosol but also at the plasma membrane and FM4–64 stained vesicles (Figure 6). Importantly, plasma membrane targeting and vesicle association seem to be, at least in part, lost for the D360N mutant, suggesting that these cellular locations are required for the death promoting function of LAZ1. Such endocytic signals and localization patterns are also reported for plant receptor-like kinases such as BR insensitive 1 (BRI1) involved in brassinosteroid (BR) signaling [36]. Receptor mediated perception of brassinolide (BL) via BRI1 involves association with the co-receptor BAK1 and endocytosis of the receptor complex. BRI1 is endocytosed constantly and independently of ligand recognition, suggesting that endosomes containing BRI1 function as intracellular signaling compartments [37]. We thus hypothesize that LAZ1 may function in the perception/transport of a death-promoting, brassinosteroid-like compound at the plasma membrane and/or such endosomal compartments in concert with BRI1, BAK1 and/or other receptors.

In conclusion, we developed a genetic screen for novel HR cell death regulators by searching for suppressors of *acd11*. This enabled us to identify the DUF300 protein LAZ1 as a regulator of certain types of HR cell death. The DUF300 domain is conserved among eukaryotes, and our initial data suggest a conserved topology for this domain in LAZ1 and in the mammalian protein TMEM34. In agreement with the structure and function of LAZ1 described here, the human homolog TMEM34 has been shown to be a tumor suppressor.

## Materials and Methods

### Plant material and growth conditions

*Arabidopsis acd11* and *acd11*/*nahG* in L*er* (*acd11-1*) and Col-0 (*acd11-2*) backgrounds, as well as L*er eds1-2*, *pad4-2,* Col-0 *eds1-2 ndr1-1 and rar1-28* have been described [4], [15]. A T-DNA insertion line of *laz1-5* (SALK_034193) was obtained from NASC (http://arabidopsis.info) and plants homozygous for the insertion were verified with T-DNA left border and gene specific primers (Table S2). Plants were grown in soil under short (8 h light/16 h dark) or long (16 h light/8 hr dark) day in chambers at 150 µE/m^2^s, 21°C and 70% relative humidity. The mutant screen and growth of F2 mapping populations were performed in a controlled greenhouse (20°C day/18°C night) with 16 h supplementary light.

### Pathogen treatments and conductivity assays

Ion leakage assays after syringe-infiltration of avirulent *Pseudomonas syringae* pv. tomato (*Pst*) DC3000 strains were performed with 2×10^8^ CFU ml^−1^ [38]. After 72 hrs, leaf disks were removed with an 8 mm cork borer and floated on distilled water for 30 min. Four leaf disks per genotype were transferred to a tube containing 4 ml of distilled water and conductivity (µS cm^−1^) measured at the indicated time points. Means and standard deviation were from 4 replicates per genotype per experiment.

### Mutant screen

Three lots of L*er acd11/nahG* seeds were mutagenized with ethyl-methane-sulphonate (EMS), di-epoxy-butane (DEB) or γ-irradiation. For EMS and DEB mutagenesis, 920–950 mg L*er acd11/nahG* seeds were pre-imbibed overnight in 0.1% KCl followed by incubation for 4 hr in either 0.74% (w/v) EMS (Sigma Aldrich Co., St Louis, MO, USA) prepared in 0.1 M sodium phosphate buffer, pH 5 with 5% DMSO, or 10 mM racemic DEB (Sigma Aldrich) in water. Subsequently, EMS-mutagenized seeds were rinsed five times in 0.1 M Na_2_S_2_O_2_ and four times in distilled water, and DEB-mutagenized seeds were washed five times in water. γ-irradiation of 300 mg *acd11/nahG* seeds was performed at the Risø Reference Laboratory with 500 Gy from a Cobalt-80 source.

M1 plants were grown in families of 125 plants, 3500 M2 plants per family were screened for BTH-resistant suppressors, giving an estimated 97% chance of recessive mutant isolation. ∼3 million M2 plants from 845 M1 pools or ∼100.000 M1 plants were scored. Putative mutants were grown on kanamycin plates, genotyped to be homozygous for *acd11* by PCR, and tested for BTH resistance in the M3 to eliminate false positives. In total, 252 suppressors from 158 families were isolated.

Analysis of inheritance and allelism sorting was performed by backcrosses to parental L*er acd11/nahG* and combinatorial crosses for 50 randomly selected suppressors. BTH-treatment of the resulting F1 progeny indicated that of 50 mutants, 35 were recessive while fifteen were dominant. Crosses to *acd11/eds1-2* and *acd11/pad4-2* verified the isolation of new *eds1* and *pad4* alleles, as well as 10 other recessive and two dominant suppressor loci (designated *laz1-12*).

### Microarray hybridization and analysis

Total RNA was isolated from three independent replicates of L*er* wild type, *nahG* and *acd11/nahG* at 0, 12, 24, 72 and 120 hr, and from *acd11/nahG/laz1-1* at 0 and 72 hr after BTH treatment. RNA was labeled and amplified according to the MessageAmp Biotin-enhanced kit (Ambion) protocol and hybridized to 51 ATH1 GeneChips after Affymetrix protocols. Data were pre-processed by RMA [39], and annotated according to the TAIR7 release of April 11, 2007. Implementation of the logit-t method in the statistical language R [40] applying two-way ANOVA instead of t-test, was used to rank genes according to their statistical significances of differential expression.

### Mapping the *Laz1* locus

L*er acd11/nahG/laz1-1* was crossed with *acd11/nahG* in Col-0 [15], and rough mapping initiated on ∼30 F2 plants homozygous for *laz1* with SSLP markers [41]. Fine mapping used 613 F2 plants with markers designed from the *Arabidopsis* polymorphism and Landsberg sequence collections (Table S2). Microarray-derived expression profiles of genes in the 200 kb interval between F20D10-A and F20M13 were analyzed for reduced expression in the *acd11/nahG/laz-1-1* mutant relative to the *acd11/nahG* background. The gene with the most reduced gene expression was sequenced.

### *In vivo* topology assays

Assays were performed as previously described [24] with modified sample buffer [50 mM DTT, 50 mM Tris-HCL, pH 7.5, 5% (w/v) SDS, 5% (v/v) glycerol, 50 mM EDTA, pH 8, 0.0025% (w/v) bromophenol blue, 1 tablet/50 ml of complete, EDTA-free protease inhibitor, Roche]. Specific primers with homologous recombination linkers (Table S2) were used for pJK90-based plasmid construction.

### Subcellular localization assays

To generate YFP-tagged LAZ1 and mutant LAZ1(D360N), full-length cDNAs were amplified by PCR and integrated into the pSAT6-YFP-C1 vector [42] using the USER cloning technique [43]. Protoplasts from *Arabidopsis* Col-0 plants were isolated by using the tape-*Arabidopsis* sandwich method adapted from Wu et al. [44]. 15 µg of YFP-LAZ1 and YFP-LAZ1(D360N) plasmids were used for each transfection experiment. After 16–18 hours incubation at room temperature and staining with FM4–64 (Sigma) for 40 min, protoplasts were imaged with a confocal laser scan microscope (Leica, SP5, Wetzlar, Germany) using a 63×/1.2W objective. For excitation of YFP and FM4–64, Argon Laser with 488 nm wavelength was used and emission bandpass filters were 500–550 nm for YFP, 580–670 nm for FM4–64.

## Supporting Information

Table S1 eds1 and pad4 alleles identified in the screen.
(0.07 MB DOC)

Table S2 List of primers.
(0.06 MB DOC)

Figure S1 laz1 suppresses acd11 cell death in the absence of nahG. Phenotypes of mature acd11/nahG/laz1-1, acd11/laz1-1, acd11 and acd11/nahG plants.
(3.15 MB TIF)

Figure S2 Expression of defense and/or cell death marker genes in acd11/nahG/laz1-1. Expression profiles of PAD4 (At3g52430), EDS1 (At3g48090), FMO1 (At1g19250), SAG13 (At2g29350), STP13 (At5g26340), WRKY6 (At1g62300), ALD1 (At2g13810), ADR1 (At1g33560) and PAD3 (At3g26830) [1]-[5] were extracted from the microarray data (Figure 2) and depicted relative to Ler wild-type controls before treatment (left). FMO1, SAG13, STP13, WRKY6, ALD1, ADR1 and PAD3 transcripts accumulated in acd11/nahG upon BTH treatment (right), and were induced in acd11/nahG/laz1-1 or background controls to a lower extent. Asterisks indicate statistical differences (*, P<0.05; **, P<0.005; ***, P<0.001) as determined by 2-way ANOVA with the factors treatment and genotype. Supplemental Reference: 1. Brodersen P, Petersen M, Pike HM, Olszak B, Skov S, et al. (2002) Knockout of Arabidopsis accelerated-cell-death11 encoding a sphingosine transfer protein causes activation of programmed cell death and defense. Genes Dev 16: 490-502. 2. Norholm MH, Nour-Eldin HH, Brodersen P, Mundy J, Halkier BA (2006) Expression of the Arabidopsis high-affinity hexose transporter STP13 correlates with programmed cell death. FEBS Lett 580: 2381-2387. 3. Robatzek S, Somssich IE (2002) Targets of AtWRKY6 regulation during plant senescence and pathogen defense. Genes Dev 16: 1139-1149. 4. Song JT, Lu H, Greenberg JT (2004) Divergent roles in Arabidopsis thaliana development and defense of two homologous genes, aberrant growth and death2 and AGD2-LIKE DEFENSE RESPONSE PROTEIN1, encoding novel aminotransferases. Plant Cell 16: 353-366. 5. Grant JJ, Chini A, Basu D, Loake GJ (2003) Targeted activation tagging of the Arabidopsis NBS-LRR gene, ADR1, conveys resistance to virulent pathogens. Mol Plant Microbe Interact 16: 669-680.
(1.03 MB TIF)

Figure S3 laz1 alleles. (A) γ-induced, one base pair deletion in laz1-1 causes a frameshift in codon 206 leading to a truncated protein with a unrelated C-terminus of 30 amino acids before a premature stop codon. (B) Structure of LAZ1 with the positions of laz1-2 and laz1-3 mutations and amplification of cDNA products with exon-specific primers (Table S2). DEB- and EMS-induced base pair changes in laz1-2 (T>A) and laz1-3 (G>A) lead to mutations in splice donor sites of introns 1 and 3, respectively. Arrows indicate primers used to investigate the effects of these mutations on LAZ1 transcripts. PCR on cDNA from acd11/nahG, laz1-2 and laz1-3 demonstrated varying sizes of laz1-2 and laz1-3 specific transcripts compared to the control (LAZ1). (C) Cloning and sequencing of PCR products indicated that laz1-2 and laz1-3 mutations resulted in partial deletions of preceding exons due to selection of abnormal upstream splice donor sites. Total PCR products (B) were purified and ligated into vector pCR-Blunt (Invitrogen). Subsequently, several clones of each mutation, as well as of the acd11/nahG control were sequenced. For laz1-2, the majority of the clones showed deletion of base pairs (bp) 86-152 of the Laz1 coding sequence, whereas clones of laz1-3 were either deleted of bp 293-316 (laz1-3a) or 208-316 (laz1-3b).
(1.81 MB TIF)

Figure S4 Alignment of LAZ1 homologs and conservation of putative endocytic motifs. Alignment of LAZ1 (At4g38360) with homologs: Arabidopsis (At1g77220, At1g23070, At5g26740, At3g05940), rice (Os_BAD61807), Physcomitrella (PpXP001785207), human (Hs_TMEM34) and mouse (Mm_OSTa). Conservation of residues is in grey scale with boxed black highest. Putative endocytic motifs are green for tyrosine based Yxxφ (x is any and φ is a hydrophobic residue). Di-leucine based motifs are red, acidic di-leucine (D, E)xxxL(L, I) pink, and acidic cluster-dileucine (DXXLL) blue. The location of LAZ1 transmembrane segments, as predicted by TMHMM v2 (www.cbs.dtu.dk/services/TMHMM/) but modified according to the results of the topology assays (Figure 5, S6), are indicated as black boxes. Non-transmembrane regions are in blue and red with red indicating cytoplasmic localization. Conservation of segments 1, 3, 4 and 5 is predicted between LAZ1 and TMEM34. The position of the laz1-4 (LAZ1(D360N)) mutation is starred.
(9.05 MB TIF)

Figure S5 Characterization of T-DNA insertion line of LAZ1 (see Table S2 for primer sequence). (A) The T-DNA integration site in laz1-5 (SALK_034193). (B) laz1-5 transcripts could not be amplified using primers P1 and P3 with 1 min elongation and 35 cycles. Equal amplification of the closest LAZ1 homolog (At1g77220) served as control (ctrl). (C) Transcripts of a fusion of laz1-5 and the T-DNA could be amplified using a T-DNA left border primer (JMLB2) and P3. (D) Transcript accumulation of LAZ1 in 6-week-old Col-0 wild-type and laz1-5 plants, as determined by qRT-PCR with primers P4 and P5 flanking the T-DNA insertion site. Expression is shown relative to the ACTIN1 gene (ACT1), mean ± SD (n = 3).
(0.94 MB TIF)

Figure S6 Disease resistance and basal defence responses in laz1-5 mutant. (A) Growth of avirulent Pst DC3000 expressing AvrRps4 on wildtype Col-0, laz1 and eds1 plants. Five-week-old plants were syringe-infiltrated with bacteria at OD600 = 0.00005 and bacterial counts per area of leaf plotted on a log scale at days 0 and 4. Bars represent means ± SD (n = 6). (B) Growth of virulent Pst DC3000 on wildtype, Col-0, laz1, eds1 or fls2 plants. Five-week-old plants were syringe-inoculated with bacteria at OD600 = 0.0001 or spray-incoculated at OD600 = 0.05 with 0.04% (v/v) Silwet L-77 (Lehle seeds). Bars represent means ± SD (n = 6, syringe inoculation; n = 4, spray inoculation). For spray inoculation, leaves were collected and surface sterilized with 70% EtOH as described [6]. Supplemental reference: 6. Zipfel C, Robatzek S, Navarro L, Oakeley EJ, Jones JD, Felix G, Boller T (2004). Bacterial disease resistance in Arabidopsis through flagellin perception. Nature. 2004 428: 764-767.
(0.99 MB TIF)

Figure S7 Topology assays for the LAZ1 human homolog TMEM34. (A) Prediction of transmembrane helices in the TMEM34 protein using the TMHMMv2 server (DTU, Lyngby, Denmark; http://www.cbs.dtu.dk/services/TMHMM/). (B) Five forms of the LAZ1 human homolog TMEM34 were C-terminally fused to the dual SUC2/HIS4C reporter to determine their orientation in membranes. These TMEM34 fusions were chosen to assess the validity of predicted transmembrane regions (see A). Predicted TM regions with high probability (>0.8) are indicated in dark grey, TM with low probability (<0.4) in light grey. (C) Growth of yeast strains harboring the five TMEM34 reporter fusions on histidinol containing medium indicates cytosolic localization of the reporter due to its histidinol dehydrogenase activity. Only fusion 1 (TMEM341-49) was unable to grow, demonstrating that the C-termini of the other 4 fusions were cytosolic. (D) Glycosylation assay on TMEM34 constructs as indicated.
(4.50 MB TIF)
