# Supplementary material for: Lazarus1, a DUF300 Protein, Contributes to Programmed Cell Death Associated with Arabidopsis acd11 and the Hypersensitive Response
Source: PLoS One. 2010 Sep 7;5(9):e12586. doi: 10.1371/journal.pone.0012586 (PMC2935358; doi:10.1371/journal.pone.0012586)
Supplement: Table S1 — eds1 and pad4 alleles identified in the screen. (0.07 MB DOC) [file pone.0012586.s001.doc]

**Supplemental Table 1**

**Table S1.** *eds1* and *pad4* alleles identified in the screen.

|  | **Allele** | **Mutagen** | **Mutation** |
| --- | --- | --- | --- |
| ***eds1*** | E111.3 | EMS | G430D |
|  | E217.2 | EMS | Q299stop |
|  | D151.1 | DEB | K478stop |
|  | E215.3 | EMS | ND |
|  | D98.1 | DEB | ND |
| ***pad4*** | E39.1 | EMS | W381stop |
|  | E77.2 | EMS | G->A in splice acceptor site of Intron 1 |
|  | D101.1 | DEB | W512R |
|  | D142.2 | DEB | C285stop |
|  | E14.2 | EMS | ND |
|  | D119.1 | DEB | ND |
|  | G56.4 | gamma | ND |

ND, not determined by sequencing
