# Supplementary material for: Lazarus1, a DUF300 Protein, Contributes to Programmed Cell Death Associated with Arabidopsis acd11 and the Hypersensitive Response
Source: PLoS One. 2010 Sep 7;5(9):e12586. doi: 10.1371/journal.pone.0012586 (PMC2935358; doi:10.1371/journal.pone.0012586)
Supplement: Table S2 — List of primers. (0.06 MB DOC) [file pone.0012586.s002.doc]

**Supplemental Table 2**

| **Table S2. List of primers** | | | | |  | |  |
| --- | --- | --- | --- | --- | --- | --- | --- |
| **Primer name** | **Sequence** |  |  | |  | |  |
| **Mapping** |  |  |  | |  | |  |
| F20M13-1 | TCT CGT AAG CAA ATC AAC GAA TAG | | | |  | |  |
| F20M13-2 | AAG ATG CGT GCG TTG ATG GAC CAA | | | |  | |  |
| F19F18-1 | GAT TAA GGA TTC TAA CTA CAT TGG | | | |  | |  |
| F19F18-2 | AAC ATT GAC GGC TCA TCT TGA TTA | | | |  | |  |
| F20D10-1 | CAG ATG TGA AGT TCT CAG ATG GAA | | | |  | |  |
| F20D10-2 | GTT TGT CTC TGA GAT TAC TAT CTC | | | |  | |  |
| **Verification of *laz1* alleles** |  |  |  | |  | |  |
| LAZ1-exon1-fwd (P1) | ATG GAT ATA TTA AAG TCT TAC CAT | | | |  | |  |
| LAZ1-exon4-rev (P2) | TTG ATA AAA CCA GGG ACT GAG CCT CCA CGG T | | | |  | |  |
| JMLB2 | TTG GGT GAT GGT TCA CGT AGT GGG CCA TCG | | | |  | |  |
| LAZ1mid-rev (P3) | CTT TGC TGG GAA TAC ATA CAG GTG | | | |  | |  |
| LAZ1-exon4-fwd (P4) | CTC AGT CCC TGG TTT TAT CAA GTT | |  | |  | |  |
| LAZ1-exon6-rev (P5) | GTC TTC AAC TGC AAG CTT TGT GCA |  | |  | |  |  |
| LAZ1h1-RT-fwd | AGT GGT GAA ATC ATT GTG GAT GAC |  | |  | |  |  |
| LAZ1h1-RT-rev | TTA CCT TCG CAT TCT AGT CCA CAA |  | |  | |  |  |
| ACT1-fwd | CGA TGA AGC TCA ATC CAA ACG A |  | |  | |  |  |
| ACT1-rev | CAG AGT CGA GCA CAA TAC CG |  | |  | |  |  |
| **Topology assay** |  |  | |  | |  |  |
| pJK92-LAZ1-fw | GTG GTT TGT TAC GCA TGC AAG CTT GAT ATC GAA ATG GAT ATA TTA AAG TCT TAC | | | | | | |
| pJK92-LAZ1-rev | TGG TCT AGA GGT GTA ACC ACT TGA GTT CTT AGG ATC TTT GGT GAT CCA TCT GCG | | | | | | |
| pJK92-LAZ1(1-54)rev | TGG TCT AGA GGT GTA ACC ACT TGA GTT CTT AGG CTT TTG CTC CTC AGG GTT CTT GTA | | | | | | |
| pJK92-LAZ1(1-158)rev | TGG TCT AGA GGT GTA ACC ACT TGA GTT CTT AGG TTG ATA AAA CCA GGG ACT GAG | | | | | | |
| pJK92-LAZ1(1-194)rev | TGG TCT AGA GGT GTA ACC ACT TGA GTT CTT AGG CCA TTT AAA CTC TCC TTC ACA GTA | | | | | | |
| pJK92-LAZ1(1-242)rev | TGG TCT AGA GGT GTA ACC ACT TGA GTT CTT AGG CGA CTT AAA TGT TAG AAA TTT AGC | | | | | | |
| pJK92-LAZ1(1-275)rev | TGG TCT AGA GGT GTA ACC ACT TGA GTT CTT AGG GC TAG TCT TCA ACT GCA AGC TTT G | | | | | | |
| pJK92-TMEM34-fw | GTG GTT TGT TAC GCA TGC AAG CTT GAT ATC GAA ATG CCT TGC ACT TGT ACC TGG | | | | | | |
| pJK92-TMEM34(1-49)rev | TGG TCT AGA GGT GTA ACC ACT TGA GTT CTT AGG CCA AGC CTT GGT GTG TAT TCC | | | | | | |
| pJK92-TMEM34(1-80)rev | TGG TCT AGA GGT GTA ACC ACT TGA GTT CTT AGG TTG TAG TTC AGG TTG TGT ATA | | | | | | |
| pJK92-TMEM34(1-99)rev | TGG TCT AGA GGT GTA ACC ACT TGA GTT CTT AGG TAT CCA ACT ATC TAA ACT GTA AAT | | | | | | |
| pJK92-TMEM34(1-171)rev | TGG TCT AGA GGT GTA ACC ACT TGA GTT CTT AGG CCT AAA CAG CAA TAC TTC TCC | | | | | | |
| pJK92-TMEM34(1-329)rev | TGG TCT AGA GGT GTA ACC ACT TGA GTT CTT AGG GGA ATC AAA GCA TGA GCC CTC | | | | | | |
