# Supplementary figures and images for: Lazarus1, a DUF300 Protein, Contributes to Programmed Cell Death Associated with Arabidopsis acd11 and the Hypersensitive Response
Source: PLoS One. 2010 Sep 7;5(9):e12586. doi: 10.1371/journal.pone.0012586 (PMC2935358; doi:10.1371/journal.pone.0012586)

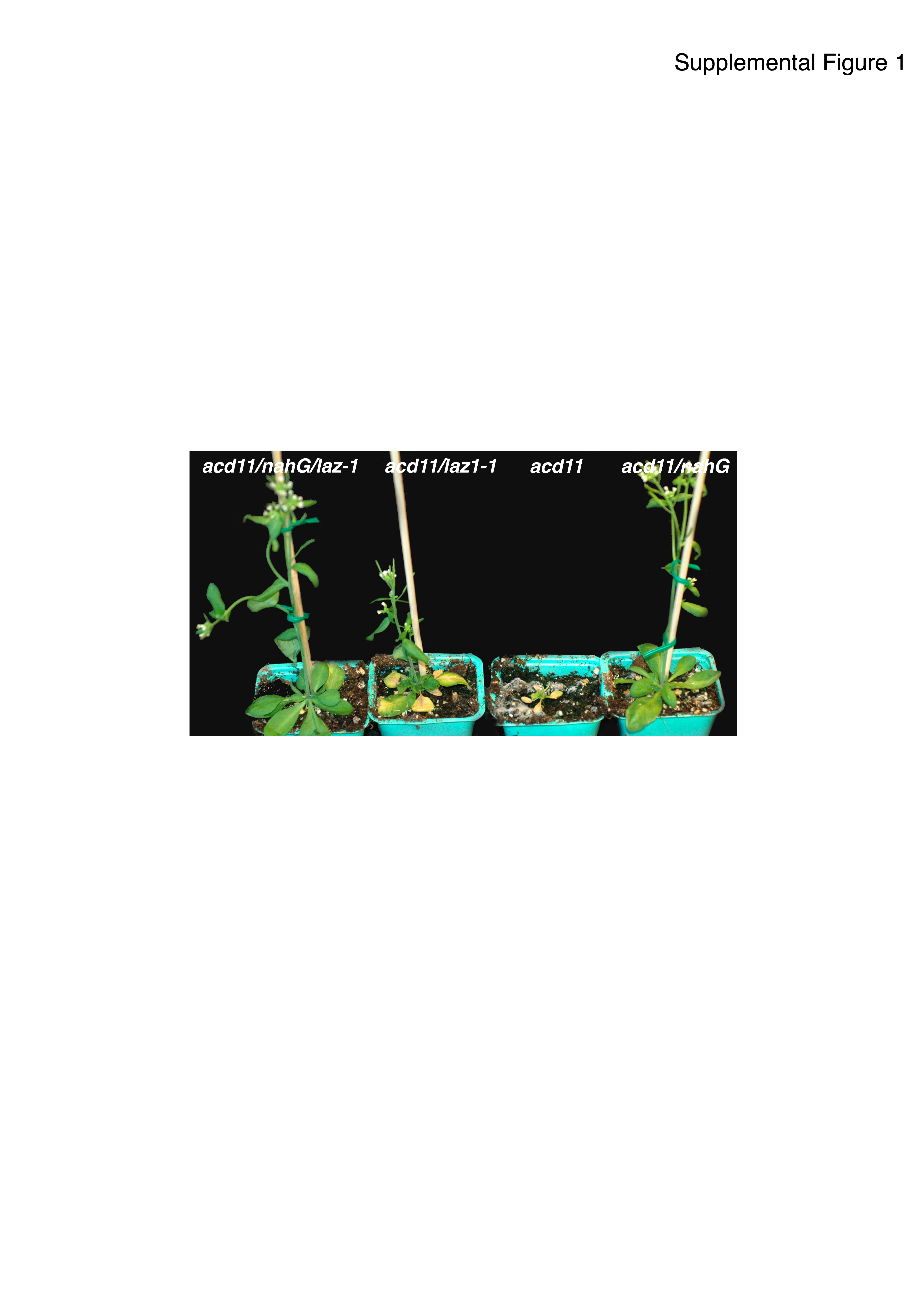

Supplement: Figure S1 — laz1 suppresses acd11 cell death in the absence of nahG. Phenotypes of mature acd11/nahG/laz1-1, acd11/laz1-1, acd11 and acd11/nahG plants. (3.15 MB TIF) [file pone.0012586.s003.tif]

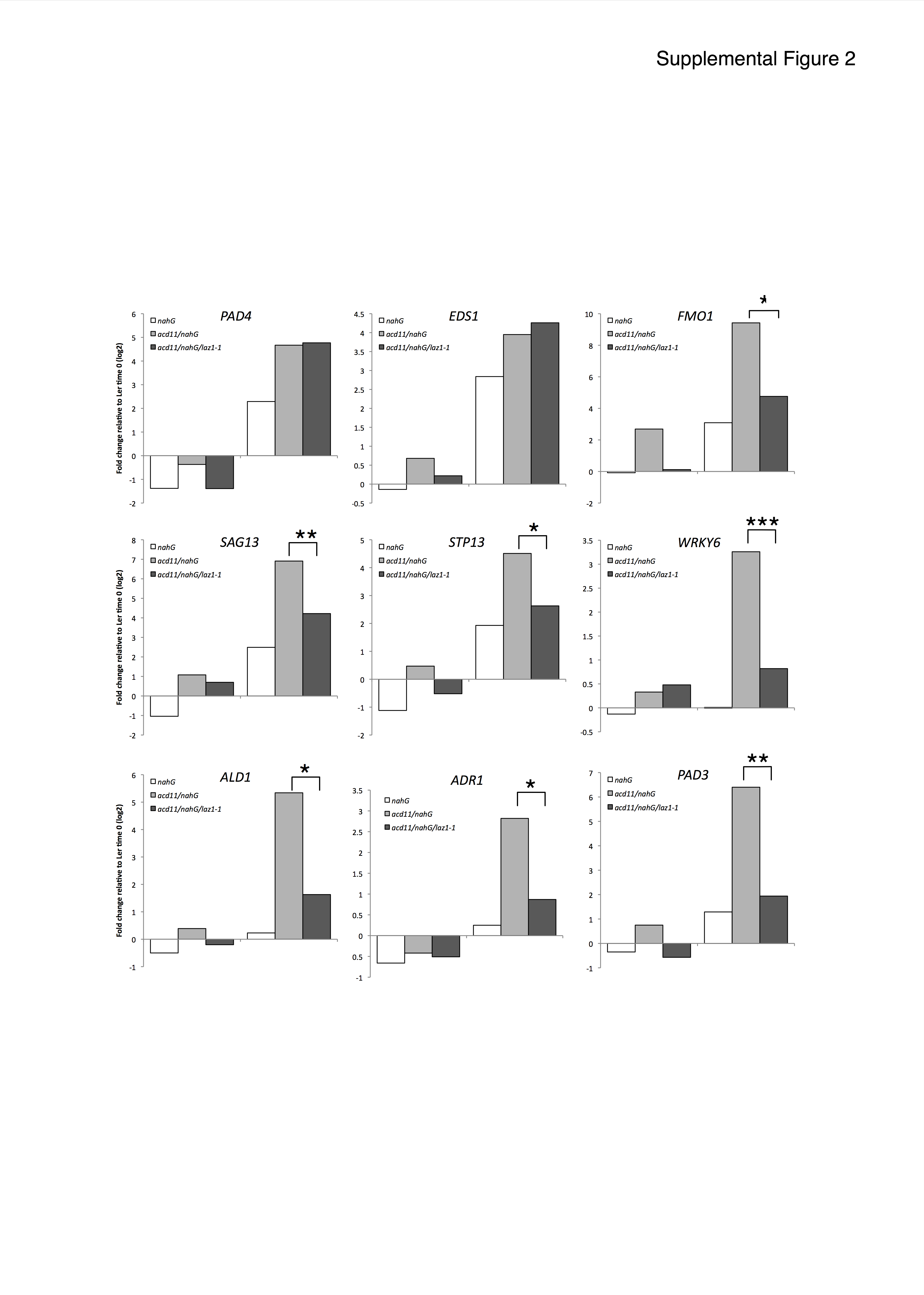

Supplement: Figure S2 — Expression of defense and/or cell death marker genes in acd11/nahG/laz1-1. Expression profiles of PAD4 (At3g52430), EDS1 (At3g48090), FMO1 (At1g19250), SAG13 (At2g29350), STP13 (At5g26340), WRKY6 (At1g62300), ALD1 (At2g13810), ADR1 (At1g33560) and PAD3 (At3g26830) [1]-[5] were extracted from the microarray data (Figure 2) and depicted relative to Ler wild-type controls before treatment (left). FMO1, SAG13, STP13, WRKY6, ALD1, ADR1 and PAD3 transcripts accumulated in acd11/nahG upon BTH treatment (right), and were induced in acd11/nahG/laz1-1 or background controls to a lower extent. Asterisks indicate statistical differences (*, P<0.05; **, P<0.005; ***, P<0.001) as determined by 2-way ANOVA with the factors treatment and genotype. Supplemental Reference: 1. Brodersen P, Petersen M, Pike HM, Olszak B, Skov S, et al. (2002) Knockout of Arabidopsis accelerated-cell-death11 encoding a sphingosine transfer protein causes activation of programmed cell death and defense. Genes Dev 16: 490-502. 2. Norholm MH, Nour-Eldin HH, Brodersen P, Mundy J, Halkier BA (2006) Expression of the Arabidopsis high-affinity hexose transporter STP13 correlates with programmed cell death. FEBS Lett 580: 2381-2387. 3. Robatzek S, Somssich IE (2002) Targets of AtWRKY6 regulation during plant senescence and pathogen defense. Genes Dev 16: 1139-1149. 4. Song JT, Lu H, Greenberg JT (2004) Divergent roles in Arabidopsis thaliana development and defense of two homologous genes, aberrant growth and death2 and AGD2-LIKE DEFENSE RESPONSE PROTEIN1, encoding novel aminotransferases. Plant Cell 16: 353-366. 5. Grant JJ, Chini A, Basu D, Loake GJ (2003) Targeted activation tagging of the Arabidopsis NBS-LRR gene, ADR1, conveys resistance to virulent pathogens. Mol Plant Microbe Interact 16: 669-680. (1.03 MB TIF) [file pone.0012586.s004.tif]

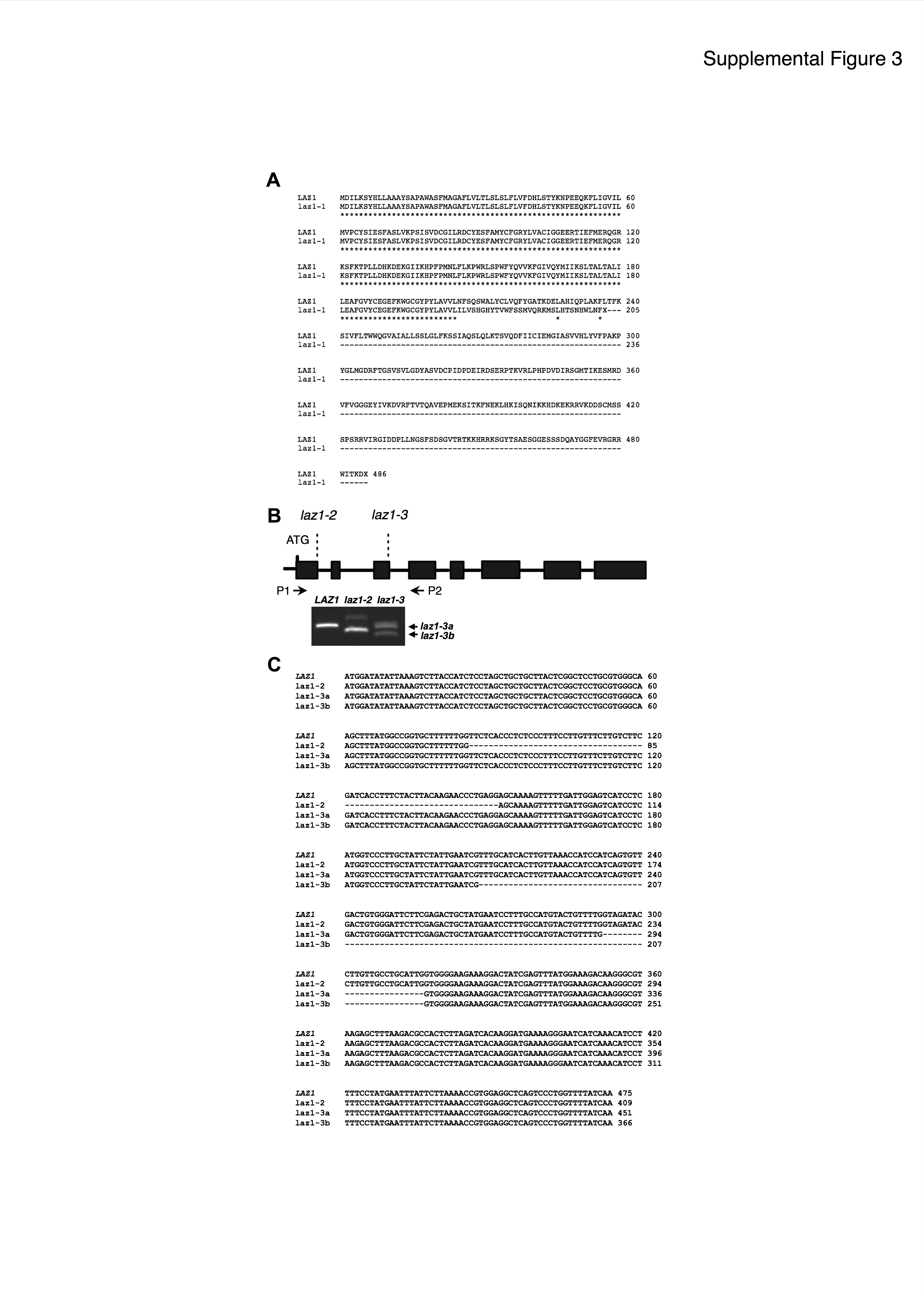

Supplement: Figure S3 — laz1 alleles. (A) γ-induced, one base pair deletion in laz1-1 causes a frameshift in codon 206 leading to a truncated protein with a unrelated C-terminus of 30 amino acids before a premature stop codon. (B) Structure of LAZ1 with the positions of laz1-2 and laz1-3 mutations and amplification of cDNA products with exon-specific primers (Table S2). DEB- and EMS-induced base pair changes in laz1-2 (T>A) and laz1-3 (G>A) lead to mutations in splice donor sites of introns 1 and 3, respectively. Arrows indicate primers used to investigate the effects of these mutations on LAZ1 transcripts. PCR on cDNA from acd11/nahG, laz1-2 and laz1-3 demonstrated varying sizes of laz1-2 and laz1-3 specific transcripts compared to the control (LAZ1). (C) Cloning and sequencing of PCR products indicated that laz1-2 and laz1-3 mutations resulted in partial deletions of preceding exons due to selection of abnormal upstream splice donor sites. Total PCR products (B) were purified and ligated into vector pCR-Blunt (Invitrogen). Subsequently, several clones of each mutation, as well as of the acd11/nahG control were sequenced. For laz1-2, the majority of the clones showed deletion of base pairs (bp) 86-152 of the Laz1 coding sequence, whereas clones of laz1-3 were either deleted of bp 293-316 (laz1-3a) or 208-316 (laz1-3b). (1.81 MB TIF) [file pone.0012586.s005.tif]

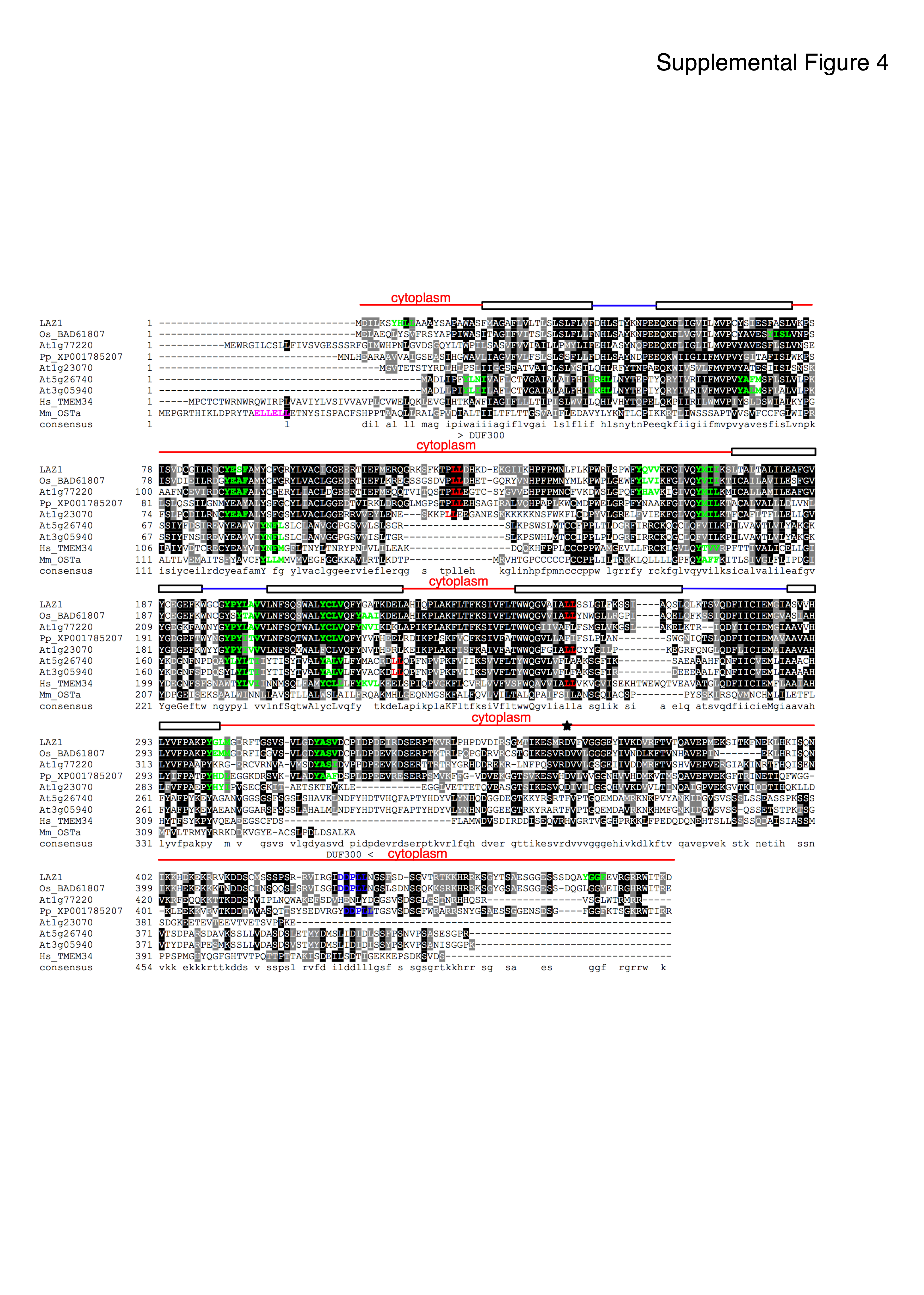

Supplement: Figure S4 — Alignment of LAZ1 homologs and conservation of putative endocytic motifs. Alignment of LAZ1 (At4g38360) with homologs: Arabidopsis (At1g77220, At1g23070, At5g26740, At3g05940), rice (Os_BAD61807), Physcomitrella (PpXP001785207), human (Hs_TMEM34) and mouse (Mm_OSTa). Conservation of residues is in grey scale with boxed black highest. Putative endocytic motifs are green for tyrosine based Yxxφ (x is any and φ is a hydrophobic residue). Di-leucine based motifs are red, acidic di-leucine (D, E)xxxL(L, I) pink, and acidic cluster-dileucine (DXXLL) blue. The location of LAZ1 transmembrane segments, as predicted by TMHMM v2 (www.cbs.dtu.dk/services/TMHMM/) but modified according to the results of the topology assays (Figure 5, S6), are indicated as black boxes. Non-transmembrane regions are in blue and red with red indicating cytoplasmic localization. Conservation of segments 1, 3, 4 and 5 is predicted between LAZ1 and TMEM34. The position of the laz1-4 (LAZ1(D360N)) mutation is starred. (9.05 MB TIF) [file pone.0012586.s006.tif]

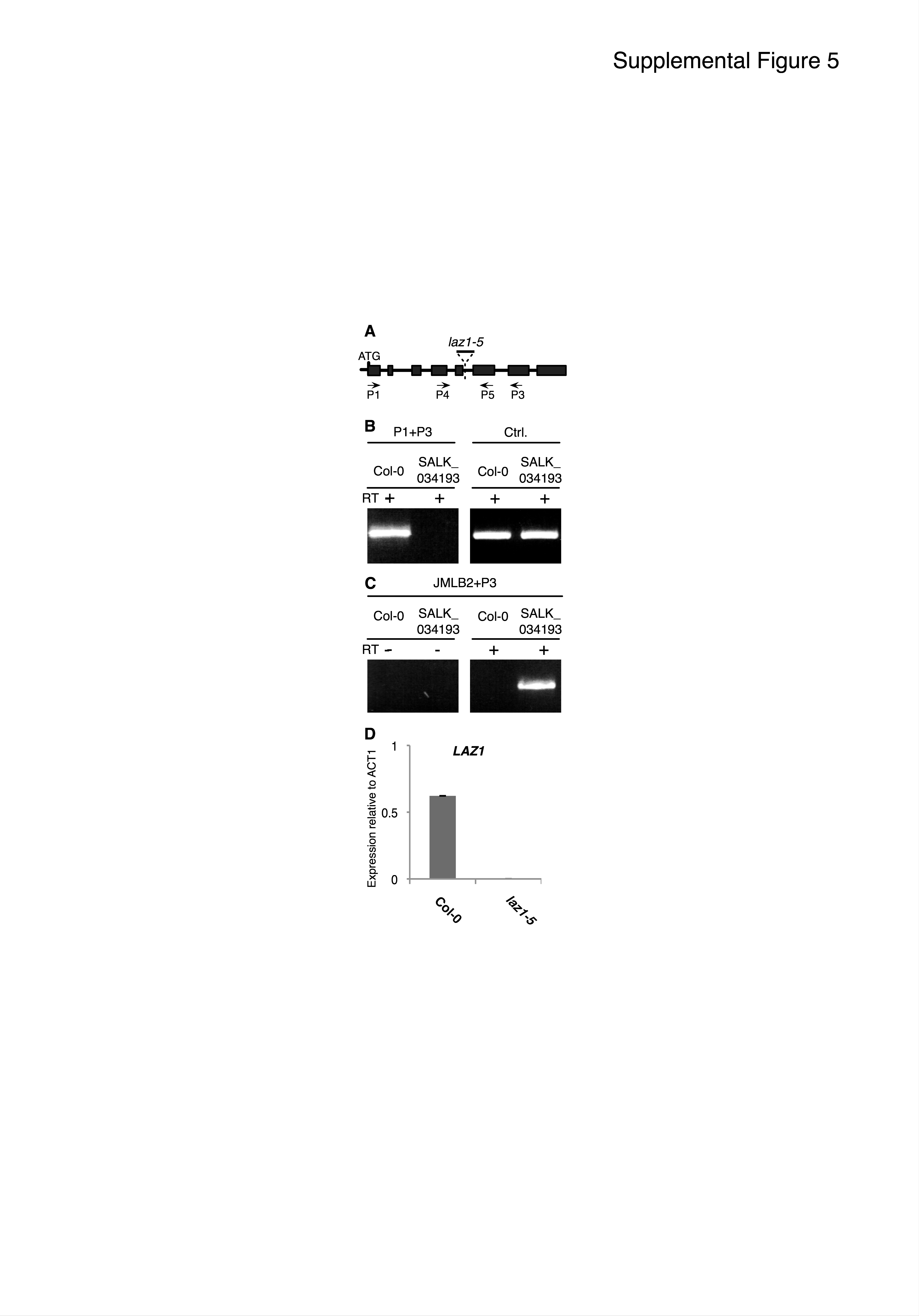

Supplement: Figure S5 — Characterization of T-DNA insertion line of LAZ1 (see Table S2 for primer sequence). (A) The T-DNA integration site in laz1-5 (SALK_034193). (B) laz1-5 transcripts could not be amplified using primers P1 and P3 with 1 min elongation and 35 cycles. Equal amplification of the closest LAZ1 homolog (At1g77220) served as control (ctrl). (C) Transcripts of a fusion of laz1-5 and the T-DNA could be amplified using a T-DNA left border primer (JMLB2) and P3. (D) Transcript accumulation of LAZ1 in 6-week-old Col-0 wild-type and laz1-5 plants, as determined by qRT-PCR with primers P4 and P5 flanking the T-DNA insertion site. Expression is shown relative to the ACTIN1 gene (ACT1), mean ± SD (n = 3). (0.94 MB TIF) [file pone.0012586.s007.tif]

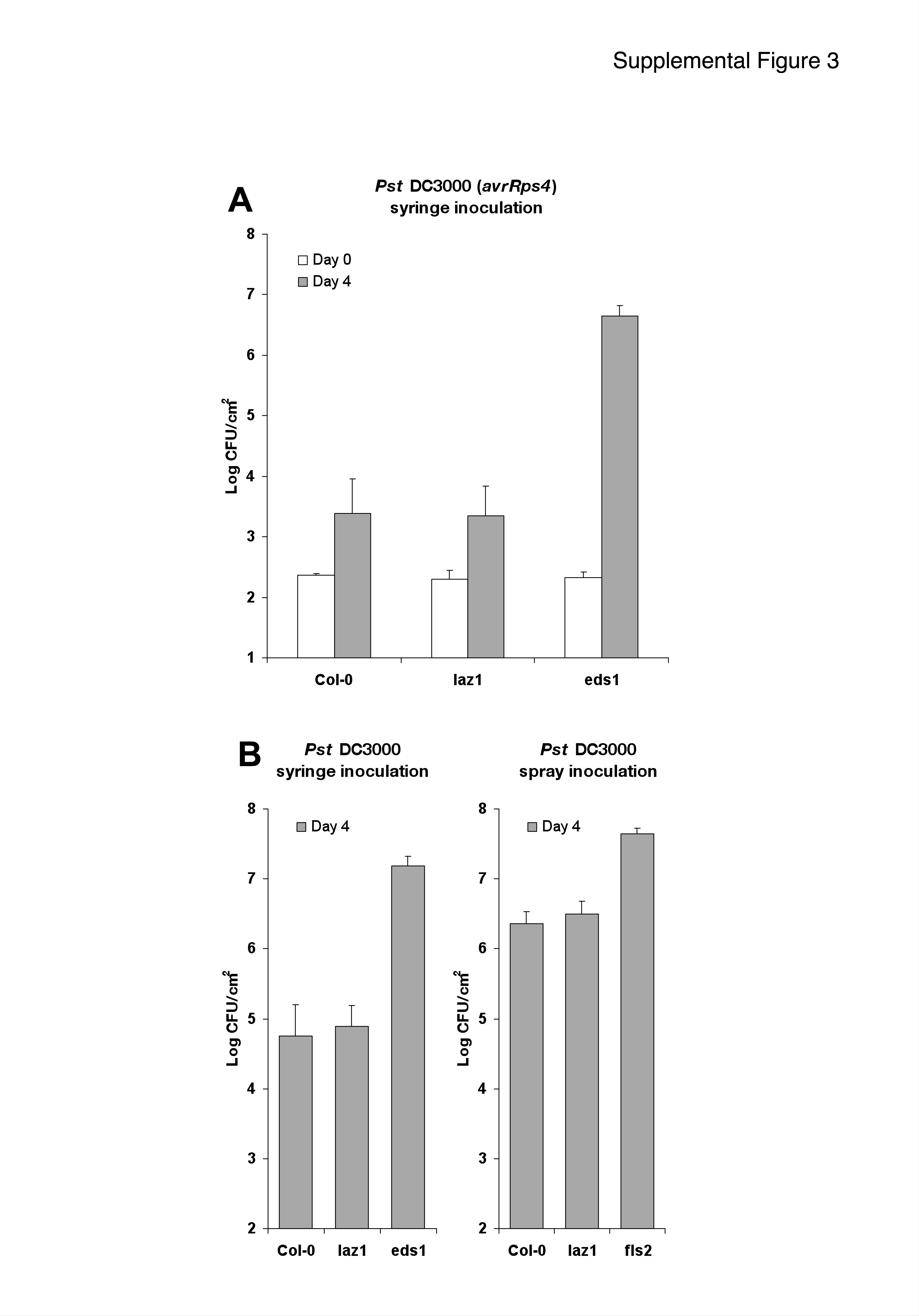

Supplement: Figure S6 — Disease resistance and basal defence responses in laz1-5 mutant. (A) Growth of avirulent Pst DC3000 expressing AvrRps4 on wildtype Col-0, laz1 and eds1 plants. Five-week-old plants were syringe-infiltrated with bacteria at OD600 = 0.00005 and bacterial counts per area of leaf plotted on a log scale at days 0 and 4. Bars represent means ± SD (n = 6). (B) Growth of virulent Pst DC3000 on wildtype, Col-0, laz1, eds1 or fls2 plants. Five-week-old plants were syringe-inoculated with bacteria at OD600 = 0.0001 or spray-incoculated at OD600 = 0.05 with 0.04% (v/v) Silwet L-77 (Lehle seeds). Bars represent means ± SD (n = 6, syringe inoculation; n = 4, spray inoculation). For spray inoculation, leaves were collected and surface sterilized with 70% EtOH as described [6]. Supplemental reference: 6. Zipfel C, Robatzek S, Navarro L, Oakeley EJ, Jones JD, Felix G, Boller T (2004). Bacterial disease resistance in Arabidopsis through flagellin perception. Nature. 2004 428: 764-767. (0.99 MB TIF) [file pone.0012586.s008.tif]

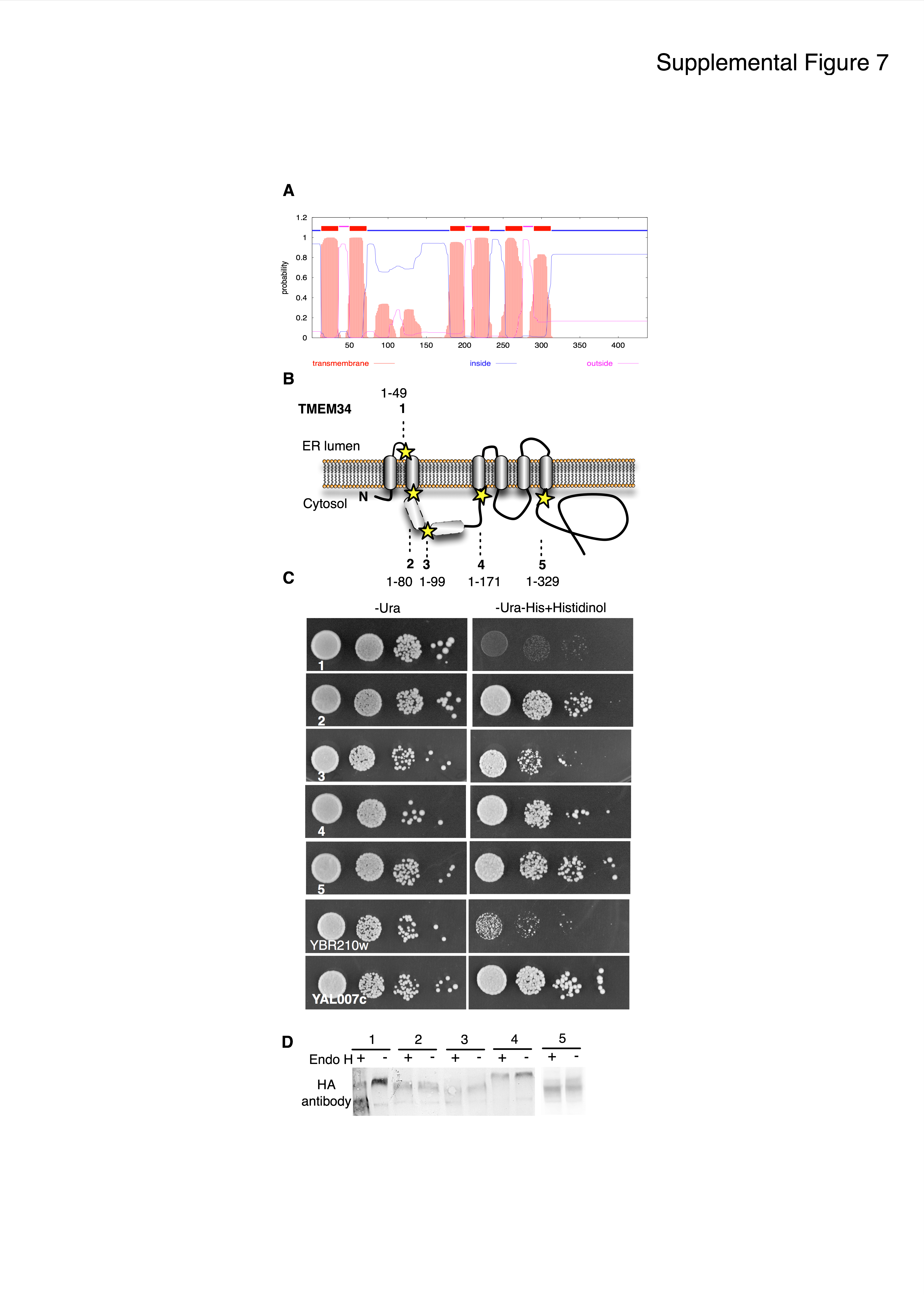

Supplement: Figure S7 — Topology assays for the LAZ1 human homolog TMEM34. (A) Prediction of transmembrane helices in the TMEM34 protein using the TMHMMv2 server (DTU, Lyngby, Denmark; http://www.cbs.dtu.dk/services/TMHMM/). (B) Five forms of the LAZ1 human homolog TMEM34 were C-terminally fused to the dual SUC2/HIS4C reporter to determine their orientation in membranes. These TMEM34 fusions were chosen to assess the validity of predicted transmembrane regions (see A). Predicted TM regions with high probability (>0.8) are indicated in dark grey, TM with low probability (<0.4) in light grey. (C) Growth of yeast strains harboring the five TMEM34 reporter fusions on histidinol containing medium indicates cytosolic localization of the reporter due to its histidinol dehydrogenase activity. Only fusion 1 (TMEM341-49) was unable to grow, demonstrating that the C-termini of the other 4 fusions were cytosolic. (D) Glycosylation assay on TMEM34 constructs as indicated. (4.50 MB TIF) [file pone.0012586.s009.tif]
